# Mob Family Proteins: Regulatory Partners in Hippo and Hippo-Like Intracellular Signaling Pathways

**DOI:** 10.3389/fcell.2020.00161

**Published:** 2020-03-19

**Authors:** Juan Carlos Duhart, Laurel A. Raftery

**Affiliations:** School of Life Sciences, University of Nevada, Las Vegas, Las Vegas, NV, United States

**Keywords:** Mob, STE20, Hippo, NDR, Tricornered, Warts, PP2A, STRIPAK

## Abstract

Studies in yeast first delineated the function of Mob proteins in kinase pathways that regulate cell division and shape; in multicellular eukaryotes Mobs regulate tissue growth and morphogenesis. In animals, Mobs are adaptors in Hippo signaling, an intracellular signal-transduction pathway that restricts growth, impacting the development and homeostasis of animal organs. Central to Hippo signaling are the Nuclear Dbf2-Related (NDR) kinases, Warts and LATS1 and LATS2, in flies and mammals, respectively. A second Hippo-like signaling pathway has been uncovered in animals, which regulates cell and tissue morphogenesis. Central to this emergent pathway are the NDR kinases, Tricornered, STK38, and STK38L. In Hippo signaling, NDR kinase activation is controlled by three activating interactions with a conserved set of proteins. This review focuses on one co-activator family, the highly conserved, non-catalytic Mps1-binder-related (Mob) proteins. In this context, Mobs are allosteric activators of NDR kinases and adaptors that contribute to assembly of multiprotein NDR kinase activation complexes. In multicellular eukaryotes, the Mob family has expanded relative to model unicellular yeasts; accumulating evidence points to Mob functional diversification. A striking example comes from the most sequence-divergent class of Mobs, which are components of the highly conserved Striatin Interacting Phosphatase and Kinase (STRIPAK) complex, that antagonizes Hippo signaling. Mobs stand out for their potential to modulate the output from Hippo and Hippo-like kinases, through their roles both in activating NDR kinases and in antagonizing upstream Hippo or Hippo-like kinase activity. These opposing Mob functions suggest that they coordinate the relative activities of the Tricornered/STK38/STK38L and Warts/LATS kinases, and thus have potential to assemble nodes for pathway signaling output. We survey the different facets of Mob-dependent regulation of Hippo and Hippo-like signaling and highlight open questions that hinge on unresolved aspects of Mob functions.

## Introduction

**FIGURE 1:**
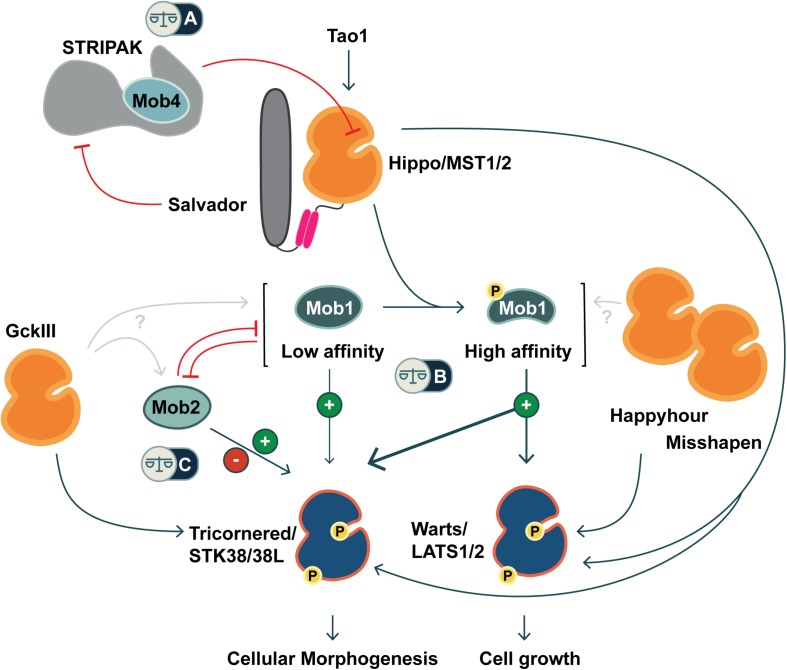
Co-regulation of Hippo and Hippo-like signaling pathways by Mob family proteins. Metazoans are equipped with two classes of NDR kinases, Tricornered- and Warts/LATS kinases. Mob family proteins have potential to balance the activities of these two classes of kinases in three ways (noted by balance icons). This pathway schematic is based on evidence from fly and mammalian literature, as discussed in the text. **(A)** As a STRIPAK component, Mob4/Phocein antagonizes Hippo/MST1/2 kinase activation. In balance with Salvador/Sav1 and Tao1, Mob4/Phocein determines Hippo/MST1/2 kinase activity. **(B)** The phosphorylation status of Class I Mob proteins alters their affinity for their NDR kinase partners. Non-phosphorylated Mob1 binds a human Tricornered-like kinase (STK38/STK38L) but not the Warts/LATS kinase (LATS1/2). Upon phosphorylation, Mob1 undergoes an activating allosteric transition that results in increased affinity for both Warts and Tricornered classes of NDR kinases. **(C)** Tricornered and Tricornered-like kinases bind both Class I and II Mob proteins. The effect of Mob2-binding to Tricornered-like kinases is unclear, with reports ascribing activating as well as inhibitory roles. Class II Mobs compete with Class I Mobs for binding to Tricornered-like kinases. In addition to Hippo, other STE20 kinases function upstream of NDR family kinases (Happyhour/MAP4K3, Misshapen/TNIK, GckIII/MST3/4), but it is not known whether they directly phosphorylate Mob family proteins.

The Mps1-binder-related (Mob) family of adaptor proteins is associated with both Hippo and Hippo-like signaling pathways. Mobs impact these pathways through interactions with both effector kinases and an inactivating phosphatase. Distinct classes of Mobs are associated with distinct complexes. Mobs are generally identified as kinase activators, due to their well-characterized ability to activate specific kinases in yeast, and to partially activate the Warts/LATS kinases (Xu et al., 1995). Warts/LATS kinases are maximally activated by Hippo kinase and are central to Hippo pathway growth control in animals. Surprisingly, Mobs are also components of a phosphatase regulatory complex known as STRIPAK, which dampens output levels for Hippo signaling (Figure 1). Altogether, Mobs appear to act as adaptors in assembling subcellular nodes that activate or inactivate Hippo. The growing network of Mobs and Mob-interacting proteins suggests that modulation of Mob levels could provide a means of controlling the availability of specific regulatory nodes within a cell or tissue (Sasaki et al., 2007; Lignitto et al., 2013; Otsubo et al., 2017; Chen et al., 2018). Regulation of Mob levels in mouse and human systems was covered recently in Gundogdu and Hergovich (2019).

The founding member of the Mob family, Mps one binder 1 (Mob1), was discovered in a yeast two hybrid screen for Monopolar spindle 1 (Mps1) kinase interacting proteins (Luca and Winey, 1998a). However, early studies of Mobs characterized them as activators of a different group of serine-threonine kinases: yeast Dumbbell former 2 (Dbf2; Komarnitsky et al., 1998) and Warts/LATS in flies and mammals (Lai et al., 2005; Hergovich et al., 2006a). Subsequently, Warts/LATS kinase and its partner Mob were defined as core constituents of the Hippo signaling pathway in flies and mammals (Hergovich, 2016; Misra and Irvine, 2018). Consistent with this function, altered regulation of, or mutations in human *mob* genes are associated with numerous cancers (reviewed in Sharif and Hergovich, 2018; Gundogdu and Hergovich, 2019).

The kinase-activating function of Mobs was supported by identification of a second group of fungal Dbf2-related kinases that are activated by dedicated Mob partners (Colman-Lerner et al., 2001; Hou et al., 2003; Maerz and Seiler, 2010). In parallel, orthologous kinases were identified in filamentous yeast, flies, and mice: Colonial temperature sensitive 1 (Cot1), Tricornered and NDR kinases, respectively (Yarden et al., 1992; Millward et al., 1995; Geng et al., 2000; Devroe et al., 2004; Hergovich et al., 2005). This second, Tricornered-like group of kinases is regulated by upstream kinases that share sequence similarity with Hippo. For this reason, we assign them to a Hippo-like kinase signaling pathway. Here, we will use “NDR kinase” as a general term to include both groups of Dbf2-related kinases.

**FIGURE 2:**
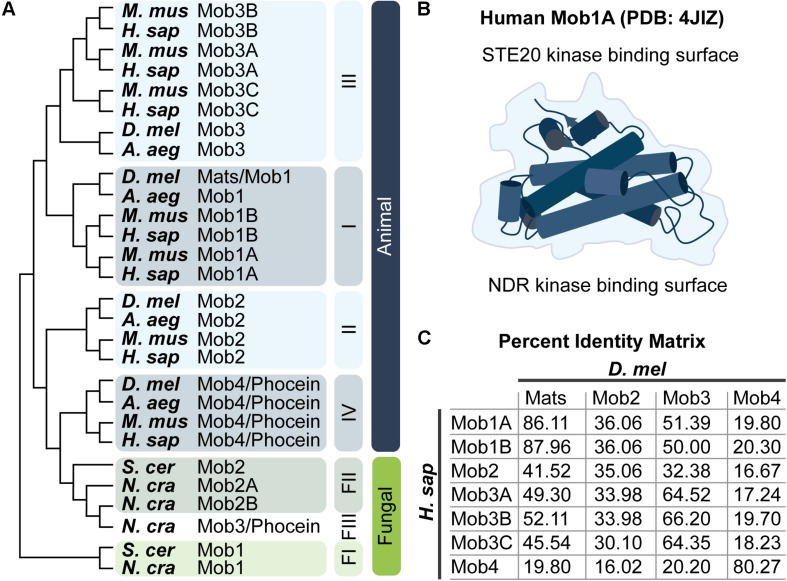
Mob family proteins are conserved from yeast to humans. **(A)** Molecular phylogenetic analysis of Mob protein sequences from the indicated species. Tree was generated using MEGA X (Jones et al., 1992; Kumar et al., 2018) as described by Hall (2013). Animal Mob proteins cluster into four distinct classes (I–IV). The relative divergence between these classes is not revealed in this limited comparison of Mobs from four animal and three fungal species – three fungal Mob classes are indicated (FI–FIII). For a thorough discussion of the evolutionary history of Mob family proteins consult the work of Vitulo et al. (2007) and Ye et al. (2009). *A. aeg*, *Aedes aegypti*; *D. mel*, *Drosophila melanogaster*; *H. sap*, *Homo sapiens*; *M. mus*, *Mus musculus*; *N. cra*, *Neurospora crassa*; *S. cer*, *Saccharomyces cerevisiae*. Refer to the Pfam database which maintains a manually curated annotation of the Mob family proteins (PF03637; El-Gebali et al., 2019). Mob family protein sequences are readily available at uniprot.org by searching for “Mob kinase activator” (The UniProt Consortium, 2019). **(B)** Artistic sketch of the human Mob1A protein (PDB: 4JIZ) reported by Rock et al. (2013). Mob proteins adopt a conserved globular fold with a core that consists of a four alpha-helix bundle, we refer to this fold as the “Mob family fold.” Binding to NDR kinases or STE20 kinases takes place on distinct Mob surfaces as indicated. **(C)** Percent identity matrix between human and fly Mob proteins.

Mps1-binder-related genes are predicted from all eukaryotes surveyed (Vitulo et al., 2007; Figure 2). Animal Mobs cluster into four classes; current evidence supports functional divergence among the four Mob classes. Only Class I Mobs are routinely designated as core components of Hippo signaling pathways (most recently in Davis and Tapon, 2019; Zheng and Pan, 2019). However, both Class I and Class II Mobs can bind NDR kinases and regulate their activity within Hippo and Hippo-like pathways. Class II Mobs may have additional adaptor functions independent of NDR kinases (Gomez et al., 2015). The more divergent Class III and Class IV Mobs have attracted less attention, but this is changing. Class IV/Phocein Mobs are components of the PP2A regulatory complex known as STRIPAK and antagonize the activation of NDR kinases, as well as others. Potential Mob functions beyond Hippo and Hippo-like pathways are reviewed by Gundogdu and Hergovich (2019) and will not be discussed here.

We begin with the structural features of the Mobs, and how they differ between classes. This leads to discussion of known interactions of Class I and Class II Mobs with NDR kinases, and then with other interacting proteins involved in Hippo and Hippo-like pathway activation. We move on to summarize functions gleaned from genetic studies, first in unicellular yeast models, and then in the fly animal model. Functional studies with mouse knockout strains and human cell lines were reviewed in depth recently (Gundogdu and Hergovich, 2019), and will not be covered here. Emerging information about STRIPAK phosphatase complex regulation of Hippo signaling is discussed last.

## Shared Structure and Distinguishing Features Between the Four Classes of Animal Mobs

How the distinct functions of each Mob class are related to their sequence divergence is poorly understood. Even the Mob family name implies a distinct function, through the reference to Mps1 kinase (Luca and Winey, 1998a), which acts in the spindle assembly checkpoint (Liu and Winey, 2012; London and Biggins, 2014). Some NDR kinases and Mobs have been implicated in spindle orientation (Frenz et al., 2000; Trammell et al., 2008; Chiba et al., 2009; Dewey et al., 2015; Yan et al., 2015), but the significance of binding interactions with Mps1 kinase remains unclear.

Generally, Mobs are single domain proteins, with an average length of 210–240 amino acids (Hergovich, 2011; Gundogdu and Hergovich, 2019). The Mob/Phocein domain adopts a conserved globular fold, based on solved structures of three classes of Mobs from multiple species: Class I Mobs from *Homo sapiens*, *Xenopus laevis*, *Mus musculus*, and *Saccharomyces cerevisiae*, a Class II Mob from *S. cerevisiae*, and a Class IV Mob from *H. sapiens* (Stavridi et al., 2003; Ponchon et al., 2004; Mrkobrada et al., 2006; Rock et al., 2013; Chung et al., 2014; Gógl et al., 2015; Ni et al., 2015; Kim et al., 2016; Couzens et al., 2017; Kulaberoglu et al., 2017; Xiong et al., 2017; Chen et al., 2018; Parker et al., 2019). This conserved structure is the Mob family fold (Figure 2B). The Mob family fold forms the NDR kinase binding surface for Class I and Class II Mobs. Whether Class III Mobs retain this tertiary structure is an open question.

Although the shared Mob/Phocein domain suggests that all Mobs might bind to NDR kinases, the evidence does not support this notion. Binding between NDR kinases and Class III or IV Mobs was undetectable in multiple independent assays (Maerz et al., 2009; Kohler et al., 2010; Ribeiro et al., 2010; Kwon et al., 2013; Xiong et al., 2017). Furthermore, NDR kinases were absent from binding partners identified both for fly Mob4 and all human Class III and IV Mobs, through proteomic surveys (Ribeiro et al., 2010; Xiong et al., 2017). Class III and IV Mobs appear to lack the capacity for stable binding to NDR kinases. In contrast, Class III and IV Mobs can physically associate with Hippo and Hippo-like kinases, in some cases as part of a STRIPAK complex. Thus, Class III and IV Mobs also contribute to regulation of Hippo signaling (Tang et al., 2014).

## Mobs as Components of NDR Kinase Activation Complexes

Many Mob gene sequences are labeled with a “kinase activator” function, due to their shared Mob/Phocein domain. This functional tag poorly represents the growing range of interactions between Mobs and other core Hippo and Hippo-like pathway core components (Mah et al., 2001; Bichsel et al., 2004; Devroe et al., 2004; Lai et al., 2005; Rock et al., 2013; Gógl et al., 2015; Xiong et al., 2018).

### Direct Mob–NDR Kinase Interactions

The kinase activator tag for Mob genes arises from their essential functions in signaling pathways involving NDR serine–threonine kinases, which are core components of Hippo and Hippo-like signaling pathways. Phosphorylation of NDR kinases by Hippo and Hippo-like kinases creates a short kinase cascade to activate downstream effectors of the pathways (Hergovich, 2013; Hergovich, 2016). At least two NDR kinase genes have been identified in sequenced eukaryote genomes, including both unicellular and multicellular fungi (Johnston and Thomas, 1982; Johnston et al., 1990; Toyn et al., 1991; Yarden et al., 1992). Plant NDR kinases are more divergent compared to those of fungi and animals, with potential for greater functional diversity, but little is known about Mob interactions with NDR kinases in plants (Kameshita et al., 2010; Katayama et al., 2012; Rademacher and Offringa, 2012; Zermiani et al., 2015; Cui et al., 2016; Xiong et al., 2016). Yeast studies have been foundational for the assignment of Mobs as NDR kinase activators.

### NDR Kinase Domains Involved in Activation and Mob Interactions

**FIGURE 3:**
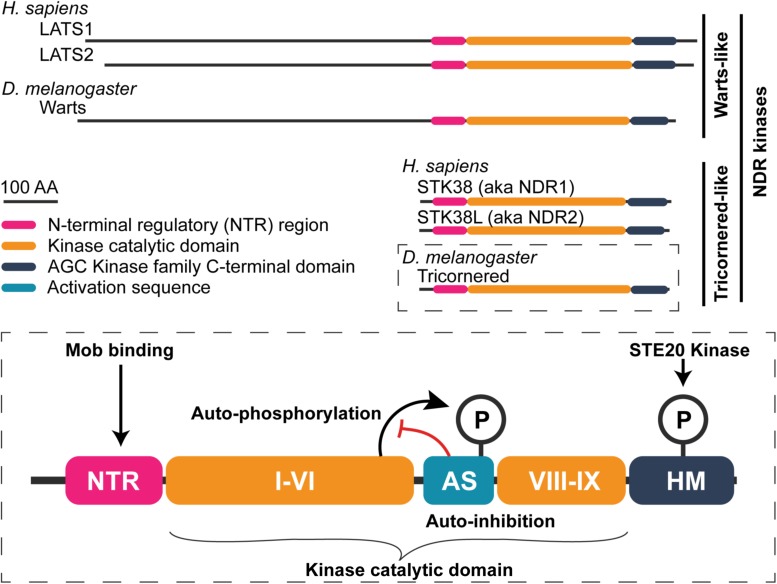
General structure of nuclear Dbf2-related (NDR) family kinases. Schematic of human and fly NDR kinases. Warts/LATS NDR kinases have a N-terminally extend domain relative to the shorter Tricornered-like kinases. The elongated N-terminus includes additional protein–protein interaction motifs (see Furth and Aylon, 2017). A detailed view of the Tricornered kinases is shown in the bottom to highlight structural features that are conserved among the NDR kinases. NDR kinases contain an NTR region to which Mob proteins bind. Within the kinase catalytic domain, between sub-domains VI and VIII, NDR kinases contain a long activation segment (AS) with auto-inhibitory function (∼30–60 amino acids in length). NDR kinase auto-phosphorylation at the AS counteracts its auto-inhibitory function. Lastly, NDR family kinases possess a hydrophobic motif (HM) in their C-terminus. Phosphorylation of the HM is essential for full kinase activation. STE20 family kinases (e.g., Hippo, Misshapen, and Happyhour in *Drosophila*) phosphorylate this motif.

Nuclear Dbf2-related kinases are members of the AGC serine–threonine protein kinase family (Hanks and Hunter, 1995; reviewed in Pearce et al., 2010; Rademacher and Offringa, 2012; Arencibia et al., 2013). Like other AGC kinases, NDR kinase activity depends on phosphorylation at two sites: the activation segment, which is conserved in all eukaryotic protein kinases, and the C-terminal hydrophobic motif (HM), which is shared by most AGC kinases. NDR kinases are distinguished by two defining features (Figure 3). The first is an extended activation segment (Millward et al., 1995; Bichsel et al., 2004), which auto-inhibits the kinase when unphosphorylated. Human NDR kinase STK38 (also called NDR1) shows increased kinase activity when that segment is mutated or deleted (Bichsel et al., 2004; Xiong et al., 2018).

The second distinguishing feature of NDR kinases is the NTR region: a structural domain that functions as the Mob-binding surface (Gógl et al., 2015; Ni et al., 2015; Kim et al., 2016; Kulaberoglu et al., 2017; Parker et al., 2019). Each of the yeast NDR kinases binds to a specific Mob that is necessary, but insufficient, for kinase activity (Komarnitsky et al., 1998; Luca and Winey, 1998a; Frenz et al., 2000; Mah et al., 2001; Colman-Lerner et al., 2001; Hou et al., 2003). The current model is that Mob binding alters the NDR kinase conformation (Gógl et al., 2015; Manning and Harvey, 2015; Vrabioiu and Struhl, 2015; Xiong et al., 2018).

Altogether, the prevailing model is that at least three regulatory inputs are required to maximally activate an NDR kinase (Figure 3). First, the NDR kinase is weakly activated by binding to a Mob. Second, the NDR kinase is phosphorylated at a conserved, C-terminal HM by Hippo or a Hippo-like kinase. Third, the weakly active NDR kinase becomes fully activated through auto-phosphorylation (reviewed in Hergovich et al., 2006b; Hergovich, 2016).

### Class I and II Mobs Bind NDR Kinases Through Overlapping but Distinct Amino Acids

The single-celled yeasts *S. cerevisiae* and *S. pombe* each encode two classes of *mob* genes and each Mob protein class has a dedicated NDR kinase binding partner. For fungi and animals, NDR kinases can be subdivided into either the Warts/LATS or the Tricornered-like class, based on their kinase domain sequence. Unlike this specificity in yeast Mob-NDR kinase binding, animal Class I Mobs are promiscuous in their association with both types of NDR kinases. For example, the fly Class I Mob, Mats, can physically associate with each of the fly NDR kinases: Warts/LATS and Tricornered (He et al., 2005a). Similarly, mammalian Class I Mobs can bind to either Warts/LATS or Tricornered-like kinases: LATS1/2 and STK38/STK38L, respectively (Bichsel et al., 2004; Devroe et al., 2004; Kohler et al., 2010; Kulaberoglu et al., 2017; Xiong et al., 2017). Partnering dynamics between a Class I Mob and an NDR kinase are conditional and selective, based on structure–function studies of murine and human Class I Mobs bound to the NTR of either a Warts/LATS or Tricornered-like kinase (Ni et al., 2015; Kim et al., 2016; Kulaberoglu et al., 2017).

Phosphorylation of Class I Mobs modulates their binding affinity for different partners (Figure 1). For example, phosphorylation of human Mob1A is necessary for detectable association with the NTR of human LATS1 kinase (Kulaberoglu et al., 2017; see also Praskova et al., 2008; Ni et al., 2015; Kim et al., 2016). In contrast, the NTR of the Tricornered-like kinase, STK38L, will readily bind to an unphosphorylated, N-terminally truncated Mob1A (Mob1A^33–216^) in isothermal titration calorimetry assays. However, the binding affinity is significantly increased when Mob1A^33–216^ is phosphorylated.

These contrasting observations may be explained by differential availability of binding sites on Class I Mobs, determined through structural studies and sequence comparisons. The NDR kinase binding surface is only partially exposed in unphosphorylated Class I Mobs (Mrkobrada et al., 2006; Ni et al., 2015; Kim et al., 2016). Full exposure of the NDR kinase binding surface is triggered by phosphorylation of highly conserved N-terminal threonine residues, through a conformational change in Class I Mobs. The importance of a phosphorylation-induced conformation change may come from the overlapping but distinct sets of amino acids that mediate binding between Mob1 and Warts/LATS kinases versus Tricornered-like kinases.

The critical amino acids have been identified for formation of mammalian Mob1-NDR kinase complexes (Kohler et al., 2010; Ni et al., 2015; Kim et al., 2016; Kulaberoglu et al., 2017). How specific amino acid contacts contribute to binding specificity was tested for a Class I Mob. One critical interaction involves a conserved histidine in Warts/LATS kinases, which forms a hydrogen bond with a class I Mob (Kulaberoglu et al., 2017). This histidine is replaced by either a phenylalanine or a tyrosine in the corresponding position of Tricornered-like kinases in yeast, flies, and humans. For human Mob1A binding to human Warts/LATS kinase, LATS1, this bond occurs between aspartic acid-63 (D63) of Mob1A and histidine-646 of LATS1. When Mob1A-D63 is replaced by a non-polar residue, valine, the resultant Mob1A-D63V mutant protein fails to bind the NTR of either fly or mammalian Warts/LATS kinases. However, Mob1A-D63V retains the ability to bind the NTR of Tricornered-like kinases, as assayed by immunoprecipitation from either fly or mammalian cells. Thus, animal Class I Mobs make distinct binding contacts with each type of NDR-kinase. The potential for differential exposure of binding sites may explain the differential binding between unphosphorylated Mob1A with either Warts/LATS kinases or Tricornered-like kinases.

Class II Mobs also have demonstrated functions as co-factors for the Tricornered-like NDR family kinases. Several studies have demonstrated this association for human Class II Mobs (Devroe et al., 2004; Chiba et al., 2009; Kohler et al., 2010; Xiong et al., 2017). A supporting result comes from an observation of Class II Mob-NDR kinase interactions in flies (He et al., 2005b). However, the functional consequences for Class II Mob binding to human Tricornered-like kinases are controversial.

Initial studies proposed that human Class II Mobs were Tricornered-like kinase co-activators (Devroe et al., 2004; Chiba et al., 2009). Devroe et al. (2004) provide three lines of evidence to support a co-activator function. First, human Mob2 directly binds human STK38/STK38L in a co-immunoprecipitation assay from 293T cells. Second, Mob2 and STK38/STK38L partly co-localize when expressed in HeLa cells. Finally, Mob2 stimulates STK38/STK38L autophosphorylation *in vitro*, in kinase assays. Consistent with these findings, Chiba et al. (2009) showed that forced expression of Mob2 in HeLa cells increases STK38 activity 5.4-fold relative to controls.

Contrasting results come from a subsequent report, where three lines of evidence suggest that human Mob2 inhibits the activity of the human Tricornered-like kinases. In this case, Mob2 antagonism is detected through competitive interactions with Class I Mobs. Kohler et al. (2010) found that Mob2 can outcompete Mob1A for STK38 binding, when assayed by immunoprecipitation from HEK293 cells (Figure 1). Strikingly, they show that Mob2 knock-down by RNAi leads to increased levels of activated STK38L, suggesting an inhibitory function. Additional experiments suggest that Mob2 can antagonize STK38/STK38L activation when Mob1A is modified with a membrane-targeting sequence in COS-7 cells. The role of Mob2 as a competitive antagonist of Mob1A remains to be tested by a comparison that uses wild-type Mob1A.

Overall, more work is needed to reconcile these opposing models for the function of Mob2 in regulating Tricornered-like kinases. It will be valuable to determine whether different cellular contexts may influence the outcomes of Class II Mob binding to Tricornered-like NDR kinases. A better understanding of the structural features and amino acid contacts that are involved in direct binding between Class II Mobs and animal Tricornered-like kinases could also be useful.

### Potential for Binding Competition Between Mobs and Other NDR Kinase Binding Proteins

For both Warts/LATS and Tricornered-like kinases, alternative binding complexes that lack Mobs have been reported. How such Mob-deficient complexes impact NDR kinase activity is emerging for Warts/LATS kinases.

#### Competition Between Mob1 and LIM Proteins for Binding to Warts/LATS Kinases

In animals, the Warts/LATS kinase N-terminus is separated from the Mob-binding NTR domain by an N-terminal extension; this domain is substantially elongated compared to the N-terminus of Tricornered-like kinases (Figure 3). Proper Warts/LATS kinase function requires the N-terminal extension, which mediates interactions with both positive and negative regulators (Yabuta et al., 2013; Furth and Aylon, 2017). Recruitment of Warts/LATS kinases to the cell membrane requires the N-terminal extension in flies and humans, primarily through association with one of several proteins: Expanded, Merlin/NF2, and Ajuba (Hamaratoglu et al., 2005; Das Thakur et al., 2010; Yin et al., 2013; Rauskolb et al., 2014; Meserve and Duronio, 2015; Yu et al., 2015; Su et al., 2017; Ibar et al., 2018). The potential for competition between Mob1 and other regulators with overlapping binding domains has been uncovered for Warts/LATS kinase association with the LIM proteins, fly Ajuba, and human TRIP6.

Ajuba inhibition of Warts/LATS kinase activity was studied in epithelial cells of fly wing primordia. Inactive Warts/LATS is enriched at adherens junctions, where it binds to its inhibitor, Ajuba (Das Thakur et al., 2010; Rauskolb et al., 2014; Meserve and Duronio, 2015). Active Warts/LATS resides more apically, where it binds to Expanded and Merlin/NF2 complexes (Hamaratoglu et al., 2005; Yin et al., 2013; Sun et al., 2015; Yu et al., 2015; Su et al., 2017). Fly Mob1 (Mats) is required for the release of Warts/LATS from Ajuba inhibition at the adherens junction and association of active Warts/LATS with more apical Expanded. The mechanism for relocation is unknown, but it requires additional proteins involved in Warts/LATS kinase activation: the scaffold protein Salvador and Hippo kinase (Sun et al., 2015).

A potential mechanism for the inhibition of Warts/LATS kinase activity comes from studies of the human LIM protein, TRIP6. Dutta et al. (2018) found that TRIP6 antagonizes LATS1/2 kinases through a direct competition with Mob1A. TRIP6 binds to the N-terminal extension at a site that overlaps the Mob binding domain of LATS1/2 kinases. LATS1/2 forms mutually exclusive complexes with either TRIP6 or Mob1, as determined by competitive binding assays. Furthermore, LATS1/2 kinase activity increased upon TRIP6 knock-out in HEK293 cells. Thus, TRIP6 antagonizes LATS1/2 activity by blocking kinase association with an activating Class I Mob.

Altogether, these studies suggest that competition between the LIM proteins, Ajuba, and TRIM6, and Class I Mobs mediates both membrane domain localization and activity of Warts/LATS kinases in both flies and human cells. However, open questions remain about the mechanisms. Does Mob binding simply release Warts/LATS kinase from the adherens junction, permitting Warts/LATS association with other Hippo pathway components in a more apical domain? Alternatively, does Class I Mob-binding have a direct role in recruiting Warts/LATS kinase to complexes at the apical membrane?

#### Evidence for Mutually Exclusive Mob1- and Beclin1-Complexes With a Human Tricornered-Like Kinase

The Mob-binding NTR domain of Tricornered-like kinases is close to the N-terminus. The absence of an N-terminally extended sequence indicates that Tricornered-like kinases are regulated in distinct ways from the Warts/LATS class. Consistent with this, Tricornered-like kinases function in autophagic pathways is associated with specific protein partners, identified through yeast-two hybrid assays (Joffre et al., 2015; Joffre et al., 2016), immunoprecipitation-mass spectrometry analysis (Klimek et al., 2019), and proximity labeling followed by mass spectrometry (Martin et al., 2019).

Beclin1 is a Tricornered-like kinase binding partner and a key regulator of autophagy. Like the Mobs, Beclin1 binds to the NTR region of the human STK38 Tricornered-like kinase. Surprisingly, both Mob1 and Beclin1 are required for STK38 function in autophagy (Joffre et al., 2015). These authors propose that STK38 binds to either Mob1 or Beclin1, but that both types of complexes have functions in autophagy. It is unclear whether Mob1 and Beclin1 compete for STK38 binding or whether partner selection is otherwise regulated. Whether other Tricornered-like kinase binding-partners can target kinase function for specific biological processes is an open question.

### Mob Proteins Interact With STE20 Kinases to Regulate NDR Kinase Activity

Hippo kinase activation of Mob-NDR kinase partners parallels the STErile20 (STE20) kinase regulation of Mob-NDR kinases, initially elucidated in yeasts. Fly Hippo kinase is a member of the STE20 kinase family, which is grouped together based on sequence similarity in their kinase domain, and on conservation of an auto-phosphorylation consensus sequence that is essential for their activation (Ling et al., 2008; Record et al., 2010). The large STE20 family is divided into the P21-Activated Kinase (PAK) and the GCK sub-families (Dan et al., 2001; Record et al., 2010; Miller et al., 2019; reviewed in Thompson and Sahai, 2015). Only the GCK sub-family kinases have been implicated in Mob or NDR kinase function.

**FIGURE 4:**
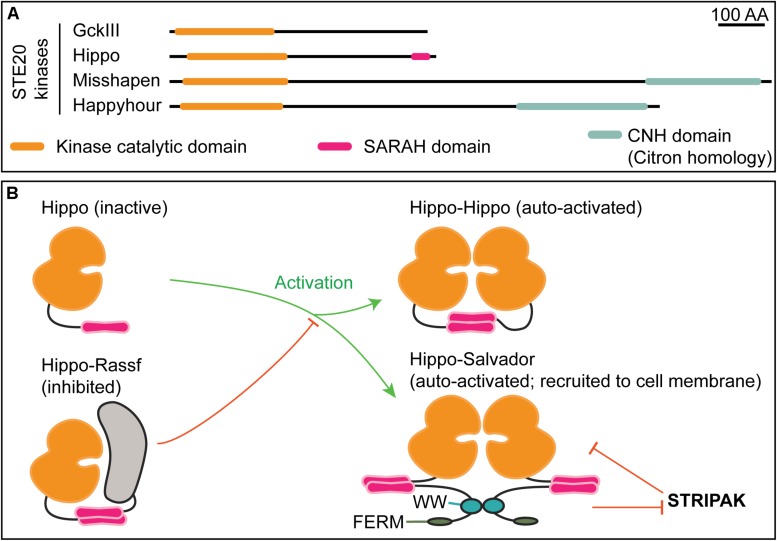
STE20 kinases have distinct domain architectures and regulatory strategies. **(A)** Schematic of select *Drosophila* STE20 kinases that have been demonstrated to phosphorylate NDR kinases. The depicted domain organization is based on annotation by UniProtKB/TrEMBL (The UniProt Consortium, 2019); the accession numbers are: Q9VEN3 (GckIII), Q8T0S6 (Hippo), Q9W002 (Misshapen), and A1ZBH7 (Happyhour). Note that Hippo is the sole STE20 kinase with an annotated SARAH (Salvador, Rassf, Hippo) domain. The function of the Citron homology domain (CNH) in NDR kinase regulation is not known. **(B)** Mutually exclusive SARAH-SARAH binding interactions provide the structural basis for positive and negative regulation of Hippo kinase activity. Formation of Hippo-Rassf antagonizes assembly of Hippo–Hippo or Hippo–Salvador complexes both of which promote full-activation via Hippo trans-autophosphorylation. When in a complex with Salvador, Hippo may be refractive to STRIPAK-mediated inactivation (Bae and Luo, 2018).

The mouse and human orthologs of Hippo kinase are the Mammalian STerile20-like kinases 1 and 2 (MST1/2). MST1 and MST2 can activate NDR kinases and have the highest sequence similarity scores to Hippo (FlyBase FB2020_01; Thurmond et al., 2019). We group them together as the Hippo kinases here. Other mammalian NDR kinase activating kinases include GckIII kinases (Stegert et al., 2005; Poon et al., 2018), MAP4Ks (Li et al., 2014, 2015; Meng et al., 2015; Zheng et al., 2015) and TAO kinases 1 and 3 (Plouffe et al., 2016). In flies, GckIII and two MAP4K-related kinases (Happyhour and Misshapen) can activate an NDR kinase (Figures 1, 4A; Li et al., 2014; Zheng et al., 2015; Poon et al., 2018). It is not clear whether MAP4K activation of NDR kinases requires an associated Mob [see Zheng and Pan (2019) for discussion].

### Mob Proteins Interact With Hippo Kinases to Regulate NDR Kinase Activity

When activated, Hippo kinases phosphorylate Class I Mob proteins, which alters the Mob’s specificity and affinity for NDR kinases (Figure 1). Hippo kinases also directly activate NDR kinases, through phosphorylation of NDR kinase HMs (Wei et al., 2007; Ni et al., 2015; Kim et al., 2016; Kulaberoglu et al., 2017; Xiong et al., 2017). In flies and mammals, Class I Mobs were initially proposed to be adaptor proteins that are required for Warts/LATS kinase activation through phosphorylation by Hippo kinases.

Mobs can directly bind to Hippo kinases in both flies and mammals. The Mob/Phocein domain has a phospho-peptide binding pocket that binds to specific phosphorylated threonines in Hippo kinase linker regions (Ni et al., 2015; Couzens et al., 2017; Xiong et al., 2017; see also Rock et al., 2013) for the analogous binding mechanism in budding yeast, *S. pombe*]. These linker residues are autophosphorylated by both Hippo and Hippo-related kinases, such as the mammalian GckIII ortholog, MST4 (Ni et al., 2015; Chen et al., 2018). Thus, Mob binding to Hippo kinases is promoted by autophosphorylation.

**FIGURE 5:**
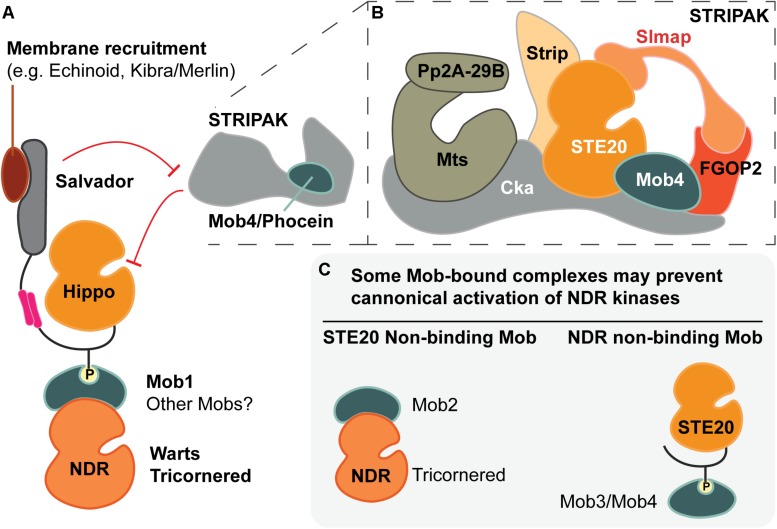
The many facets of Mob regulatory function. **(A)** Metazoan Mob1 proteins have well-established roles as allosteric co-activators of NDR kinases and as scaffold proteins that bridge STE20 kinases (e.g., Hippo) to their phosphorylation targets, the NDR kinases. Together with other scaffolds, such as Salvador, these activation complexes are recruited to specific subcellular locations. In canonical Hippo signaling, the PP2A phosphatase of the STRIPAK complex inhibits Hippo via dephosphorylation. The most divergent fly Mob (Mob4/Phocein) is required for STRIPAK activity. **(B)** Cartoon of the STRIPAK complex adapted from Ribeiro et al. (2010), Zheng et al. (2017) and Tang et al. (2019). **(C)** Speculative Mob-dependent strategies of NDR kinase signaling output. Some classes of Mob proteins appear to bind STE20- or NDR-kinase binders, but not both. Such interactions yield Mob-bound complexes that may not allow formation of canonical activation complexes as pictured in part **(A)**.

Mob-driven assembly of Class I Mob-NDR kinase-Hippo kinase ternary complexes is thought to be a critical step in NDR kinase activation. The Mob structure could facilitate complex formation, in that the phospho-Hippo binding pocket of a Class I Mob is located opposite to the NDR kinase binding surface (Figure 2B; Kim et al., 2016). This structural arrangement would link Hippo kinases to both phosphorylation substrates: The Mob and its NDR kinase binding partner (Figure 5A; Ni et al., 2015). In support of this view, deletion of the Mob-binding linker region of the human Hippo kinase, MST2, blocks it from phosphorylating Mob1.

Whether other Hippo-related kinases bind to or phosphorylate Mobs is an open question. NDR kinases can be activated by MAP4K- and GckIII-group GCK kinases, but it is unknown whether Mobs are involved (Gundogdu and Hergovich, 2019; Zheng and Pan, 2019). It remains unclear whether Mobs can generally recognize and bind to these Hippo-related kinases through phospho-threonines in the kinase linker domains.

One study has raised the question of whether stable Mob1-Hippo kinase binding is essential for Mob1 phosphorylation or Warts/LATS kinase activation by Hippo kinases (Kulaberoglu et al., 2017). To test the requirement for Class I Mobs in Hippo kinase binding to its substrate NDR kinase, Kulaberoglu et al. (2017) generated a human Mob1A^K104E/K105E^ mutant that fails to bind fly or human Hippo kinases. However, Mob1A^*K*104E/K105E^ retains the ability to bind to either class of NDR kinase when assayed by co-immunoprecipitation in human or fly cells. Expression of Mob1A^*K*104E/K105E^ rescues lethality in *mats* mutant flies, which lack the single Class I Mob, and restores normal expression of Warts target genes in fly wing primordia. These results suggest that STE20 family kinases that lack phospho-peptide motifs recognized by Mobs may still contribute to NDR kinase activation.

The possibility that Mobs can be bypassed in Hippo activation of NDR kinases might indicate that recruitment of Hippo-like kinases and Warts-Mob1 complexes to overlapping sub-cellular domains is sufficient to overcome the loss of stable Mob1-Hippo-like kinase binding (as presented in Yin et al., 2013; Su et al., 2017). Alternatively, brief, or unstable interactions between Mob1A^*K*104E/K105E^ and Hippo kinase may permit sufficient Warts kinase activity for pathway responses *in vivo*, even though such binding cannot be detected by standard assays. More work is needed to reconcile distinct interpretations for Class I Mob function in NDR activation complex assembly (compare models proposed by Kulaberoglu et al., 2017; Manning and Harvey, 2015; Ni et al., 2015; Vrabioiu and Struhl, 2015; Bae and Luo, 2018; Zheng and Pan, 2019).

Moving beyond Class I Mobs, all human Mobs, except Mob2, bind to a MST1-derived phospho-peptide sequence (Couzens et al., 2017); and share analogous phospho-binding pockets. Binding relies on three phosphate-coordinating basic residues [lys-153, arg-154 and arg-157 in human Mob1A, which are not conserved in human Mob2 (Rock et al., 2013; Ni et al., 2015; Couzens et al., 2017)]. Furthermore, human Mob4 binds the GCK-III STE20 kinase, MST4, through a binding interface that is similar to the human Mob1–MST1 complex; binding is also dependent on MST4 linker phosphorylation (Chen et al., 2018). Taken together, these findings suggest that most classes of Mobs are general STE20 kinase-binders. However, the Class III and IV Mobs appear not to bind NDR kinases (Kohler et al., 2010; Ribeiro et al., 2010; Kwon et al., 2013; Xiong et al., 2017), raising questions about the functions of Class III or Class IV Mob interactions with a STE20 kinase.

Human Mob4 may contribute to Warts/LATS kinase regulation indirectly by blocking Mob1 association with Hippo kinase (Chen et al., 2018). Mob4 outcompetes Mob1, in a dose-dependent manner, for binding to the human Hippo kinase, MST1 in a co-immunoprecipitation assay from PANC-1 cells. More work is needed to critically assess the possibility that Mobs contribute to STE20 signaling output through regulation of phosphorylation-target selection and/or sub-cellular localization. Additional scaffold proteins are involved in animal NDR kinase activation; they are briefly surveyed in the next section.

One feature that distinguishes Hippo kinases from other GCK kinases in the STE20 family is a SARAH domain, named after three proteins that have it: SARAH (Scheel and Hofmann, 2003). Hippo kinases bind their regulatory partners, Salvador and Rassf family proteins, through SARAH-SARAH domain binding (Sanchez-Sanz et al., 2016; Bae et al., 2017; Bae and Luo, 2018; Cairns et al., 2018). In flies, Salvador-Hippo binding leads to kinase activation (Figure 4B), whereas Rassf-Hippo binding results in kinase inhibition (Polesello et al., 2006). Thus, the SARAH domain provides one structural basis for regulation of Hippo kinase activity. The regulatory features that control the activity of other GCK kinases in NDR kinase activation are less understood.

### Both Mobs and Scaffold Proteins Contribute to Pathway Activation Through Distinct Interactions in Different Sub-Cellular Domains

The NDR kinase name includes “Nuclear” due to an initial observation of nuclear localization when the human Dbf2-related kinase, STK38, was over-expressed (Millward et al., 1995). However, both Warts/LATS and Tricornered-like kinases move between nuclear, cytoplasmic, and/or cortical locations (Devroe et al., 2004; Emoto et al., 2004; He et al., 2005b; Hergovich et al., 2005; Horne-Badovinac et al., 2012; Yin et al., 2013; Sun et al., 2015). It is now appreciated that NDR kinase subcellular localization is tightly regulated and linked to signaling output of Hippo and Hippo-like pathways.

Early studies pointed to Mobs as regulators of human NDR kinase localization (Hergovich et al., 2005, 2006a). When human Mob1 is targeted to the membrane, both Tricornered-like and Warts/LATS kinases are localized to the membrane and potently activated (Hergovich et al., 2005, 2006a), a result reproduced in flies (Ho et al., 2010). We previously discussed a potential role for Mob1 in subcellular localization of Warts/LATS. Whether animal Mobs generally target NDR kinases to specific subcellular locations, as has been documented in yeast (Rock et al., 2013), is still unclear.

In flies and mammals, Salvador and Furry family proteins have been characterized as scaffolds that play essential roles in NDR kinase activation (Cong et al., 2001; Kango-Singh et al., 2002; Tapon et al., 2002; Chan et al., 2005; Chiba et al., 2009). Furry-related proteins are conserved from yeast to humans, while Salvador family proteins appear to lack clear homologs in yeast (Gruneberg et al., 2001; Du and Novick, 2002; Nelson et al., 2003; Rock et al., 2013; Nagai and Mizuno, 2014). We will focus on the animal scaffolds in this section, to emphasize context-dependent regulatory interactions during assembly of the NDR kinase activation complex. Notably, Salvador and Furry proteins, together with Mobs, promote the assembly of animal Hippo and Tricornered-like kinase activation complexes in specific sub-cellular locations.

#### Salvador Proteins Promote Activation of Animal Warts/LATS Family Kinases

Salvador proteins are required for the activation of Warts/LATS NDR kinases *in vivo* (Kango-Singh et al., 2002; Tapon et al., 2002; Pantalacci et al., 2003), and are considered core components of Hippo signaling. Activation of Warts/LATS kinases occurs near the plasma membrane; thus, subcellular localization of Warts/LATS NDR kinases and other activation complex components provides a potential step for pathway regulation. Substantial evidence supports the role of Salvador in recruitment of Hippo and MST1/2 kinases to specific domains of the plasma membrane. The emerging model hinges on Hippo/MST1/2 activation and presents a multi-functional view of Salvador function. On the one hand, Salvador promotes Hippo/MST1/2 activation via kinase auto-phosphorylation (Ni et al., 2013; Bae et al., 2017). On the other hand, Salvador antagonizes negative regulators of Hippo/MST1/2 kinase activity (Bae et al., 2017).

Salvador proteins are non-catalytic, with three defining domains: a FERM (4.1, Ezrin, Radixin, Moesin) binding domain, two WW domains, and a C-terminal SARAH domain. Salvador proteins localize to two distinct cortical regions. In flies, this cortical localization is mediated, in part, through association with the FERM domain-contain protein Merlin and the IgG-domain cell adhesion molecule Echinoid (Chishti et al., 1998; Yu et al., 2010; Yue et al., 2012; Su et al., 2017). Echinoid adhesive complexes form at the apical margin of adherens junctions, which are the most apical of the lateral junctions in insects (Fulford et al., 2018). Echinoid recruits Salvador to the cortex adjacent to adherens junctions (Yue et al., 2012; Sun et al., 2015). Alternatively, Merlin, in association with Kibra, recruits Salvador to the apical membrane (Yu et al., 2010; Su et al., 2017). Each of these membrane domains represent sites of Hippo pathway activation (Sun et al., 2015; Su et al., 2017).

A compelling model for the mechanism by which Salvador activates Hippo kinases is supported by structural and biochemical studies of fly and human Salvador (Bae et al., 2017; Cairns et al., 2018; reviewed in Bae and Luo, 2018). In this model, Salvador participates in hetero-tetrameric complexes consisting of two Salvador proteins and two Hippo/MST1/2 kinases (Figure 4B). Formation of this hetero-tetramer promotes kinase trans-autophosphorylation.

While formation of these structures may stabilize Hippo/MST1/2 kinase interactions, they are not essential for kinase activation *per se*. Purified MST1/2 kinases are fully active *in vitro*, suggesting they can self-activate independently of co-factors. *In vivo*, the requirement for Salvador may be bypassed in some contexts, when independent activating kinases are present. Consistent with a bypass mechanism, the GCK sub-family kinase, Tao-1, phosphorylates and activates Hippo in *Drosophila* eye and wing primordia. In these tissues, loss of Tao-1 leads to overgrowth phenotypes that are associated with decreased Hippo kinase activation (Boggiano et al., 2011; Poon et al., 2011). If the Salvador dependence can be bypassed, why is Salvador thought to be required for Hippo pathway activation?

One possibility is that Salvador has additional functions in activation of Hippo kinases, some of which may be context-dependent. Consistent with this interpretation, formation of fly Salvador-Hippo complexes blocks accessibility of the Hippo SARAH domain for binding to RASSF, a Hippo kinase inhibitor (Polesello et al., 2006; Ribeiro et al., 2010). However, evidence from mammalian cells supports both positive and negative regulation of Hippo kinase activity by mammalian RASSF family proteins (Avruch et al., 2009; Richter et al., 2009; Ni et al., 2013; Aruna et al., 2017). It may be that the differing results from the fly and mammalian systems are simply due to different pathway configurations between the cell types examined (see discussion in Rawat and Chernoff, 2015).

A second potential function for Salvador is to block an inactivating phosphatase. The STRIPAK complex antagonizes Hippo kinase activity by removal of activating phosphates. Human Salvador1 binds to, and inhibits, the catalytic subunit of PP2A of the STRIPAK complex in 293FT cells (Bae et al., 2017). A third possibility is that Salvador may be essential to enrich active Hippo at specific cellular locations, such as the apical or sub-apical membrane domains of epithelial cells (Yin et al., 2013; Su et al., 2017).

More work is needed to understand context-dependence for Salvador functions. Beyond epithelia, Hippo-like kinases function upstream of NDR kinases in other polarized cell types such as neurons, but the sub-cellular distribution of Salvador regulators, like Merlin, are less understood (Emoto et al., 2004, 2006; Reddy and Irvine, 2011). Broadening our exploration of these pathways in distinct tissue contexts will illuminate apparently conflicting data on Hippo regulation.

#### Animal Furry Family Proteins Promote the Activation of Tricornered-Like Kinases

Furry family proteins are essential components of Tricornered-like NDR signaling systems in specific cellular contexts, from yeast to humans (Cong et al., 2001; Du and Novick, 2002; Hirata et al., 2002; Gallegos and Bargmann, 2004; Chiba et al., 2009; Norkett et al., 2019; Goto et al., 2010). Binding between Furry and Tricornered-like kinases is reported for fly and mammalian proteins (Chiba et al., 2009; Fang et al., 2010), but little is known about the structural features that make Furry proteins essential to Tricornered-like activity. Furry proteins are large, ranging 2000–3000 residues in length, and contain five to six conserved regions (Nagai and Mizuno, 2014). Of these, the N-terminal-most region contains Armadillo repeats that may be a platform for protein–protein interactions with multiple partners (Tewari et al., 2010). However, aside from this domain, the function of the remaining conserved sequences is unknown (Nagai and Mizuno, 2014).

In HeLa cells, Furry is required for chromosomal alignment during metaphase (Chiba et al., 2009). Furry is required for activation of the Tricornered-like kinase, STK38, in both genetic and biochemical assays; and this function is enhanced by Mob2. Furry most-likely forms a complex with Mob2-STK38, based on co-immunoprecipitation. Notably, Furry-Mob2 binding is only detected under conditions of pathway hyperactivation, when cells are treated with a phosphatase inhibitor. Conversely, Mob2-STK38 binding appears to be constitutive and insensitive to STK38 phosphorylation status. In dividing HeLa cells, STK38 kinase activity levels are cell-cycle dependent and reach their maximum at metaphase. At this stage, Furry co-localizes extensively with spindle-microtubules. Together, these findings raise the following critical questions: Does STK38 activation require the formation of a Furry-Mob2-STK38 ternary complex, or is STK38 activation a pre-condition for complex assembly? In either case, it is unclear how the hippo-like kinase, MST2, associates with these proposed complexes and whether Furry restricts STK38 function to the mitotic spindle.

## Distinct Mobs Form Dedicated Activation Complexes for Their NDR Kinase Partners to Regulate Distinct Physiological Events in Fungi

Budding and fission yeast, as well as filamentous fungi, are each equipped with two classes of NDR family kinases. Each kinase is independently regulated by dedicated activation complexes, which consist of a Mob family protein, a STE20-like kinase, and a molecular scaffold. Following activation, each of the two classes of fungal NDR kinases, in complex with their exclusive Mob partners, participates in distinct cell biological roles. Generally, the fungal Warts/LATS kinases are key regulators of cell cycle progression while the Tricornered-like kinases play essential roles in cellular morphogenesis. A filamentous fungal species, the mold *Neurospora crassa*, has four Mobs, three of which interact with dedicated NDR kinase partners, and a fourth Mob3/Phocein, which does not (Maerz et al., 2009). In filamentous fungi, Mob3/Phocein functions in the hyphal growth phase and in sexual development (Maerz et al., 2009; Fu et al., 2011), as do other proteins in the fungal STRIPAK complex (Bloemendal et al., 2012). The striking parallels between fungal and animal STRIPAK-Hippo antagonism in regulation of actin cytoskeletal organization and formation of cellular protrusions such as filopodia or dendrites are reviewed by Kuck et al. (2019).

### Fungal Warts/LATS Kinases Play Key Roles in Cell Cycle Progression

The prototypical NDR kinase, *S. cerevisiae* Dbf2, is a Warts/LATS-type kinase identified in a genetic screen for defective cell division, detected by the shape of connected daughter-cell pairs (Johnston and Thomas, 1982; Johnston et al., 1990). Subsequently, a paralogous Warts/LATS kinase, Dbf20, was identified (Toyn et al., 1991). Dbf2/20 are central components of the MEN, a signaling pathway that promotes disassembly of the mitotic spindle, chromosome de-condensation, and cytokinesis (Luca and Winey, 1998b; reviewed in Bardin and Amon, 2001; Hotz and Barral, 2014). Dbf2/20 activation is necessary to exit mitosis and relies on a dedicated multi-protein complex comprised of: Cdc15, a Ste20-like protein kinase; Mob1, the prototypical Mob; and Nud1, a scaffold protein. Mutation of any one of these genes results in mitotic exit failures.

In *S. pombe*, Sid2, also in the Warts/LATS NDR kinase group, functions in an analogous signaling pathway: The SIN (Hou et al., 2000; Salimova et al., 2000; Bardin and Amon, 2001). This signaling network promotes cytokinesis. Activation of Sid2, the terminal kinase in the SIN, relies on inputs from Sid1, a Ste20-like protein kinase; Mob1, a Mob family protein, and Cdc11-Sid4 a scaffold complex. The *S. pombe* Cdc11 is the homolog of the *S. cerevisiae* Nud1 scaffold protein (Tomlin et al., 2002). Loss of function in *sid2* or its co-activators results in the formation of multinucleated cells due to failed cytokinesis following DNA synthesis and mitosis (Krapp and Simanis, 2008). The core kinase cassettes of the MEN and SIN are largely conserved in flies and mammals, where they function as essential components of the Hippo pathway (Hergovich and Hemmings, 2012).

### Fungal Tricornered-Like Kinases Play Key Roles in the Regulation of Cellular Morphogenesis

The first Tricornered-like NDR kinase, Cot-1, was identified in the filamentous fungus, *Neurospora crassa* (Yarden et al., 1992). In *N. crassa*, *cot-1* mutants exhibit morphogenetic defects associated with impaired hyphal tip elongation and excessive branching. Homologous Tricornered-like NDR kinases were subsequently identified in *S. cerevisiae* and *S. pombe*: Cbk1 and Orb6, respectively (Fulvia et al., 1995, 1998; Nasr et al., 1996; Racki et al., 2000). Like Cot-1, Cbk1 and Orb6 have demonstrated roles in the regulation of cellular morphology. In *S. cerevisiae*, Cbk1 is a central kinase in the RAM pathway (Bidlingmaier et al., 2001; Nelson et al., 2003). Activation of Cbk1 requires inputs from Kic1, a Ste20-like protein kinase; Mob2, a Mob family protein, and Tao3/Pag1, a Furry like protein scaffold (reviewed in Weiss et al., 2002; Nelson et al., 2003). Loss of function in Cbk1 or its co-activators leads to defects in cell shape changes associated with mating, including a reduction in apical growth and impaired formation of polarized mating projections (Bidlingmaier et al., 2001). In *S. pombe*, Orb6 functions in the analogous MOR (Hachet et al., 2012). Activation of Orb6 requires inputs from Nak1, a STE20-like protein kinase; Mob2, a Mob family protein, Mor2, a Furry family scaffold protein. Orb6 loss of function or loss of other MOR network components results both in defective actin polarization and polarized cell growth.

### Fungal Mob1 and Mob2 Proteins Have Dedicated NDR Kinase Binding Partners

In each of the model fungi, *S. cerevisiae*, *S. pombe*, and *N. crassa*, the two classes of NDR family kinases have dedicated Mob family binding partners (Maerz et al., 2009; Parker et al., 2019). This differs from Mob-NDR partnering dynamics in animals where there is genetic and biochemical evidence for Mob-NDR promiscuity. How is partner selectivity determined in these fungal systems?

The structural features that ensure specific Mob-NDR partner interactions in fungi are emerging (Aharoni-Kats et al., 2018). Studies of *S. cerevisiae* Mob-NDR complexes show that Mob1 and Mob2 each have a tri-peptide motif that confers binding selectivity, called kinase restrictor motifs (Parker et al., 2019). These motifs are within the NDR kinase binding-surface and have the sequence Arg–Gly–Glu in Mob1, and Lys–Tyr–Val in Mob2 (Parker et al., 2019). Indeed, exchange of this kinase restrictor motif between the two yeast Mobs is sufficient for the two chimeric Mobs to exchange their NDR kinase binding partners in a pull-down assay from cell lysates. Additionally, the chimeric Mobs bind to non-cognate NDR kinases with high affinity. Thus, the kinase restrictor motif determines the selectivity of Mob binding to a specific NDR kinase in *S. cerevisiae*.

Intriguingly, the *S. cerevisiae* Mob1 kinase restrictor motif is conserved among Class I Mob family proteins across a wide range of eukaryotes including invertebrates, vertebrates, and plants. Conversely, the *S. cerevisiae* Mob2 kinase restrictor motif is not conserved in animal Class II Mobs. Perhaps divergence from *S. cerevisiae* Mob1 kinase restrictor motif sequence underlies their differences in binding to Warts/LATS kinases versus Tricornered-like kinases.

## Mob-NDR Kinase Signaling in *Drosophila* Hippo and Hippo-Like Pathways

While the work carried out in fungi provides an indispensable foundation for understanding Class I and Class II Mobs, the expansion of the animal *mob* gene family in multicellular organisms is associated with added complexity in the functions of Class I and Class II Mobs. In fungi, exclusivity in Mob-NDR kinase partnering allows for independent regulation of distinct NDR kinases with distinct functions in fungal physiology. In contrast, some animal Mobs exhibit promiscuity with respect to their NDR binding partners. The molecular and cell-biological implications for NDR co-regulation by shared Mob binding partners are only beginning to be appreciated (see Gundogdu and Hergovich, 2019). How this impacts cell and tissue function will depend on the combination of Mobs and alternative NDR kinase co-regulators within that cell type. To highlight the functional outputs that are controlled by Mob-dependent pathways, we focus on physiological and morphological studies in *Drosophila*, where genetic methods have facilitated *in vivo* analyses.

### An Expanded Set of Co-activator Proteins Regulate the Activation of Drosophila NDR Kinases

The fly genome encodes a single kinase from each NDR kinase subfamily: Warts and Tricornered. However, there is one representative from each of the four animal Mob classes. The four fly genes encoding Mob-family proteins are named *mats*, *mob2*, *mob3*, and *mob4* (Ye et al., 2009). As for the mammalian Mobs, the Class I fly Mob, Mats, binds to both Warts and Tricornered kinases. In contrast, Mob2 has only been reported to bind to Tricornered kinase (He et al., 2005b). Consistent with the observed physical interactions, Mats functions in fly Hippo signaling as an essential Warts co-activator. Tests for genetic interactions with *tricornered* support a role for Mats as a Tricornered kinase co-activator (Geng et al., 2000; He et al., 2005b). Conversely, the cell biological role of Mob2 is unclear.

Flies have an expanded set of GCK subfamily kinases, relative to fungi, with nine genes. Of these, *hippo*, *happyhour*, *misshapen*, and *gckIII* are known to function upstream of fly NDR kinases (Staley and Irvine, 2012; Li et al., 2014; Zheng et al., 2015; Poon et al., 2018). Lastly, the *Drosophila* genome encodes two scaffold proteins that are required for NDR kinase activation: *salvador* and *furry* (Cong et al., 2001; Kango-Singh et al., 2002; Tapon et al., 2002). The following sections discuss the fly pathways that involve these genes, and the physiological processes that the pathways control.

### Fly Warts NDR Kinase Is Central to the Highly Conserved Hippo Growth-Restrictive Pathway

Since the seminal identification of the Hippo pathway, multiple upstream regulators and downstream targets have been identified that implicate the core Hippo signaling pathway, comprised of Hippo kinase, Warts kinase, Mats (Mob1), and Salvador, as a key regulator for a diverse set of cellular and physiological functions, including stem cell maintenance, cellular differentiation, epithelial cell mechano-transduction, cytoskeletal dynamics, cell migration, organogenesis, tissue homeostasis, and pathology (reviewed in Staley and Irvine, 2012; Matsui and Lai, 2013; Yu and Guan, 2013; Irvine and Harvey, 2015; Fallahi et al., 2016; Chen et al., 2019; Sahu and Mondal, 2019; Snigdha et al., 2019). In this section, we focus only on the specific studies that identified the genes and illuminated our understanding of the signaling pathways.

Many components of the animal Hippo signaling pathway were identified through forward genetic screens in flies (reviewed in Kim and Jho, 2018b; Gokhale and Pfleger, 2019). In 1993, Bryant et al. (1993) sought to identify tumor suppressor genes using a genetic mosaic screen, which identified *warts* based on the “spectacular outgrowths from the body surface” that formed from *warts* mutant epithelial cells. Then in 1995, two groups independently cloned the *warts* gene, one group naming the gene *lats* for its identified tumor suppressor phenotype (Justice et al., 1995; Xu et al., 1995), leading to identification of *warts* as a Dbf2-related kinase. Since then, many groups used genetic mosaic screens to identify growth suppressors through recovery of mutant alleles that produced epithelial tissue overgrowth phenotypes. The first Hippo signaling pathway gene identified through such screens was *salvador*, independently isolated by two groups (Kango-Singh et al., 2002; Tapon et al., 2002). Overgrowth of *salvador* mutant epithelial cells arises from both increased cell proliferation and reduced apoptotic cell death.

The *hippo* gene, named due to the enlarged heads that result from presence of *hippo* loss of function cells in mosaic developing head epithelia, was identified by five groups (Harvey et al., 2003; Jia et al., 2003; Pantalacci et al., 2003; Udan et al., 2003; Wu et al., 2003). Cells that lack Hippo kinase, similar to those that lack Warts kinase or the Salvador scaffold, show increased cellular proliferation with reduced levels of the *Drosophila* inhibitor of apoptosis, DIAP1, and increased levels of the G1–S cell cycle regulator, Cyclin E (Kango-Singh et al., 2002; Tapon et al., 2002). Genetic experiments placed these genes in the same growth restricting, or tumor suppressor pathway. Furthermore, Hippo, Warts, and Salvador proteins physically bind to each other in biochemical assays. Altogether, these data indicated that Hippo, Warts, and Salvador are essential components of a STE20 kinase-NDR kinase signaling pathway.

The Mob component of the Hippo pathway was discovered by Lai et al. (2005), who identified the Class I Mob, Mats, as an essential co-activator of Warts kinase. Like other fly Hippo pathway genes, *mats* mutant cells in mosaic developing tissues lead to enlarged adult tissues. Together, these studies established the fly Hippo-Warts-Mats-Salvador intracellular signaling pathway, which restricts tissue growth.

Shortly after the discovery of Mats, the transcription factor Yorkie (named for homology to the mammalian Yes associated protein or YAP) was identified as a critical target of Warts kinase (Huang et al., 2005). When a substantial proportion of developing adult structures consist of cells that lack *yorkie* function, a miniaturized fly forms, with otherwise normal morphologies and viability. Conversely, Yorkie overexpression leads to striking tissue overgrowth. These are the opposite phenotypes obtained for similar experiments with Warts kinase, or its activators, where large numbers of cells lacking the upstream pathway genes give rise to overgrown adult tissues. Genetic experiments placed *yorkie* downstream of *warts* and biochemical assays demonstrated that Yorkie is a Warts kinase phosphorylation target (Huang et al., 2005). It was later shown that Warts-dependent phosphorylation of Yorkie generates a binding site for 14–3–3 proteins (Dong et al., 2007). Phosphorylated Yorkie is therefore sequestered in the cytoplasm, which prevents its transcriptional regulatory activity (Dong et al., 2007; Oh and Irvine, 2008; Zhang et al., 2008; Oh and Irvine, 2009; Ren et al., 2010).

Several studies suggest a non-transcriptional role for Yorkie. In epithelial cells of the developing wing and eye, and in follicular epithelia of adult ovaries, a small fraction of the total cellular Yorkie is at the apical cortex (Oh et al., 2009; Fletcher et al., 2018; Xu et al., 2018). This apical Yorkie pool promotes activation of non-muscle myosin II in developing wing epithelial cells, independently of Yorkie’s nuclear role as a DNA-binding transcriptional regulator (Xu et al., 2018). Furthermore, cells mutant for *warts* show strong enrichment of cortical Yorkie. Although this finding suggests that Warts kinase limits Yorkie apical localization, it does not address whether the Warts kinase needs to be fully activated by a combination of Mob and STE20 kinase actions. In this regard, it will be illuminating to determine whether Mats is required for Yorkie apical localization.

### Fly Tricornered Kinases Acts in a Hippo-Like Pathway Controlling Cellular Morphogenesis

Tricornered is emerging as a genetic regulator of cellular morphogenesis in several fly tissues and its regulatory partners are coming to light. Overall, studies of the fly Tricornered kinase have lagged behind those of the fly Warts/LATS kinase. The *tricornered* gene was identified in 1976; *tricornered* mutant cells produce split and morphologically aberrant wing hairs (Ferrus, 1976; Gubb and García-Bellido, 1982; Vinson and Adler, 1987). The Adler lab determined that *tricornered* encodes a NDR kinase (Geng et al., 2000). The *furry* gene was later identified in a genetic screen for wing hair polarity mutants and showed a genetic interaction with *tricornered* (Cong et al., 2001). Wing epithelial cells that are mutant for either *tricornered* or *furry*, produce multiple, split, and aberrantly shaped wing hairs, a phenotype associated with defects in actin bundling (Geng et al., 2000; Cong et al., 2001; He et al., 2005b; Fang and Adler, 2010).

Fly Tricornered regulation by Mob proteins is poorly understood. Potential genetic interactions between *tricornered* and each of the four fly *mob* genes have been probed by assessing their effects on wing hair formation. Forced expression of a non-activatable *tricornered^*T*453*A*^* transgene in wing epithelial cells produces multiple hairs (He et al., 2005b; Fang and Adler, 2010). The T453A mutation abolishes the HM threonine that is phosphorylated by Hippo and GckIII (He et al., 2005b; Emoto et al., 2006; Poon et al., 2018; see also Millward et al., 1999). When *tricornered^*T*453*A*^* overexpressing wing epithelial cells are also hemizygous for any one of the four *mob* genes, they more frequently produce aberrant wing hairs, and the defects are more extreme. These data implicate multiple Mob family proteins in Tricornered NDR kinase pathways. However, to date, Mats and Mob2 are the only fly Mobs demonstrated to physically bind Tricornered (He et al., 2005a), and the links between these observations and the underlying cell biology are not clear. Subsequently, studies in the pupal eye imaginal disc implicated Mob2 as a regulator of photoreceptor cell morphogenesis (Liu et al., 2009). In this tissue, RNAi mediated knock-down of *mob2* results in defective rhabdomere formation and pigment cell differentiation. Although these studies implicate Mob2 function in cellular morphogenesis, the underlying mechanism is unclear, because Tricornered function was not investigated in this tissue.

Additional genetic evidence that fly Mob2 is required for Tricornered activation comes from studies of the larval neuromuscular junctions, where synaptic contacts form between motor neuron axons and muscle. Campbell and Ganetzky (2013) showed that *mob2* is required for normal development of neuromuscular junctions. Each junction involves multiple neuronal contacts called synaptic boutons. Neurons with reduced *mob2* function produce broad axonal extensions with more synaptic boutons relative to controls. The presence of a heterozygous mutation in *tricornered* worsened the *mob2* junction defects. Presence of a heterozygous mutation in *warts* however gave no alteration. Subsequently, RNAi depletion of *tricornered* in neurons gave defects resembling the *mob2* synaptic bouton defects (Natarajan et al., 2015). These investigators detected reduced presynaptic levels of the Actin regulator, WASP (Wiskott–Aldrich Syndrome Protein) in *tricornered* mutant larvae, accompanied by reduced *wasp* mRNA. This latter result raises the question of whether Tricornered may regulate downstream transcription factors but provides no evidence for whether such regulation may be direct or indirect. Taken altogether, the data from studies of neurons in flies support a role for Tricornered in regulation of the actin cytoskeleton and roles for both Tricornered and Mob2 in formation of synaptic boutons. Parallels have been observed in mammalian neurons, where NDR kinases phosphorylate proteins involved in endocytosis and vesicle trafficking, substrates required for dendrite growth (Ultanir et al., 2012; Leger et al., 2018).

Tricornered and Furry also have related functions in the regulation of larval neuronal morphogenesis (Emoto et al., 2004, 2006). *Drosophila* sensory neurons of the larval ectoderm form large arbors of dendrites. *Tricornered* function is required for both normal dendrite morphogenesis and the tiled organization of dendritic branches, where the dendritic arbors from different sensory neurons are organized in non-overlapping patterns (Emoto et al., 2004, 2006; reviewed in Jan and Jan, 2010; Parrish, 2016). Neurons mutant for either *tricornered* or *furry* have excessive and overlapping dendritic branches (Emoto et al., 2004, 2006; Koike-Kumagai et al., 2009). Subsequently, Norkett et al. (2019) have shown that Tricornered, in partnership with Furry, regulates neurite growth through phosphorylation of the kinesin-like protein Pavarotti, in a mechanism that inhibits microtubule sliding (Del Castillo et al., 2015; Norkett et al., 2019). It will be exciting to see whether Mob proteins participate in this process, and if so, which classes of Mobs are involved.

Hippo kinase functions biochemically and genetically upstream of both Tricornered and Warts kinases to regulate sensory neuron dendritic tiling and maintenance, respectively (Emoto et al., 2006). Overexpression of transgenic wild-type *tricornered* rescues tiling defects of *hippo* mutant neurons. This result supports a role for Tricornered downstream of Hippo kinase but raises the question of whether another STE20 kinase can bypass the requirement for Hippo kinase to fully activate Tricornered. Alternatively, elevated levels of partially activated Tricornered, perhaps with a Mob partner, might be sufficient to mediate repulsive signaling that blocks dendrites from extending into the vicinity of a different neuron. To understand context-dependent Hippo and Hippo-like pathway activities, it will be important to explore the relationships between pathway components in this peripheral nervous system context. If Hippo is the activating kinase for both Tricornered and Warts in sensory neurons, the regulation of NDR kinase switching, as well the potential for NDR kinase competition for Hippo kinase, could be investigated productively with the same assays.

Results from other organs implicate additional STE20 kinases in genetic Tricornered pathways. Both *tricornered* and *furry* regulate egg elongation during oogenesis (Horne-Badovinac et al., 2012). When follicular epithelial cells are mutant for either gene, they form round eggs instead of the normal ellipsoid eggs. Follicular epithelial cells require Tricornered for anisotropic egg elongation into an ellipsoid shape, whereas Hippo appears to be dispensable (Meignin et al., 2007; Polesello and Tapon, 2007; Yu et al., 2008; Yan et al., 2011). Instead, a MAP4K kinase, Misshapen, is required. When most follicular epithelial cells are mutant for *misshapen*, the resultant round eggs resemble those formed by *tricornered* or *furry* mutant epithelial cells (Horne-Badovinac et al., 2012; Lewellyn et al., 2013). Thus, Misshapen is part of an egg elongation regulatory system; as such, it is a candidate activating kinase for Tricornered in this system. However, this potential pathway relationship has not been tested, either *in vivo* by genetics or *in vitro* in cultured cells (reviewed in Gates, 2012; Cetera and Horne-Badovinac, 2015).

A third STE20 kinase, GckIII, is a bonafide activator of Tricornered kinase in the morphogenesis of larval tracheae (Figure 1; Song et al., 2013; Poon et al., 2018). However, Mob function in tracheal morphogenesis was not addressed. These results highlight the value of proteomic and forward genetic screens for uncovering new contexts where alternative NDR kinase activation pathways should be investigated critically. Such studies will be needed to assess whether Mobs are required for full NDR kinase activation when the NDR kinase is activated by a STE20 kinase that differs from Hippo.

### Emerging Roles for the Class IV Mobs as Components of the Highly Conserved STRIPAK Complex

In this section we discuss emerging roles for the most divergent class of Mob family proteins – the Phocein or Class-IV Mobs. In flies and mammals, Mob4/Phocein proteins have been identified as components of the highly conserved STRIPAK complex. Filamentous fungi have a Phocein-like Mob, which shares developmental phenotypes with genes encoding STRIPAK components, which suggests that Class IV/Phocein proteins have conserved function across multicellular eukaryotes (Maerz et al., 2009; Fu et al., 2011; Bloemendal et al., 2012).

As a component of STRIPAK, Mob4/Phocein is a negative regulator of fly Hippo signaling. It remains unclear whether the Mob4/Phocein proteins act exclusively via STRIPAK or whether, like Class I and Class II Mobs, they can also function as direct regulators of NDR family kinases. The two classes of NDR family kinases share many of their regulators, it will be challenging to unravel the mechanisms that ensure balanced signaling through each of the two kinases is adequately coordinated for their functions in any specific cell type. Pursuing the role of Mob4/Phocein function in relation to that of STRIPAK is a compelling entry into this question, because of its potential to restrict NDR kinase signaling via the regulation STE20 kinases (e.g., Hippo, Misshapen, GckIII, and their mammalian homologs). Consistent with this notion, multiple mammalian STE20 kinases of the GCK II and III subfamilies interact with STRIPAK components (Sugden et al., 2013; Hwang and Pallas, 2014).

#### The STRIPAK Complex Is a Negative Regulator of Hippo Signaling

Phosphorylation of NDR kinases is required for their full activation. *In vivo*, this activation is dampened by protein phosphatases (Hergovich et al., 2006b). The first evidence that PP2A decreases NDR kinase activity (Millward et al., 1999) took advantage of a potent phosphatase inhibitor, okadaic acid, which preferentially inactivates PP2A (Takai et al., 1995; Favre et al., 1997). Both increased kinase activity and increased phosphorylation of the human Tricornered-like kinase were observed following treatment of COS-1 cells with okadaic acid (Millward et al., 1999). Subsequent studies of yeast and fly NDR kinases reported similar effects in response to okadaic acid treatment, indicating that negative regulation of NDR kinases by phosphatase activity is conserved from yeast to humans (Mah et al., 2001; Chan et al., 2005; Lai et al., 2005; Hergovich et al., 2006a; Koike-Kumagai et al., 2009; Zheng et al., 2017).

Proteomic studies linked PP2A regulation of Hippo signaling pathways to STRIPAK, a highly conserved multimeric protein complex that includes PP2A, a Mob/Phocein protein, and STE20 kinases of the GCK subfamily (Figure 4B; Glatter et al., 2009; Goudreault et al., 2009; Hyodo et al., 2012; Sugden et al., 2013; Madsen et al., 2015; Shi et al., 2016). Moreover, studies in flies and mammals have shown that STRIPAK is a potent negative regulator of Hippo signaling (Ribeiro et al., 2010; Couzens et al., 2013; Zheng et al., 2017; Gil-Ranedo et al., 2019; Tang et al., 2019). In this role, PP2A in the STRIPAK deactivates Hippo kinases, which in turn leads to decreased Warts/LATS kinase activation. Notably, fly Mob4 and the STRIPAK complex are required for normal formation of synaptic boutons at fly neuromuscular junctions, a process regulated by Tricornered and Mob2 (Schulte et al., 2010; Neisch et al., 2017).

Our current understanding of the structural basis for STRIPAK complex assembly is limited, but exciting new details are emerging (Tang et al., 2019). Tang and colleagues examined interactions between major mammalian STRIPAK components and propose a “two-arm” model of STE20 kinase recruitment to the STRIPAK complex. In this model, the proteins STRIP1 and SLMAP (Strip and Slmap in flies), bind MST2 and MST4 kinases (Hippo and GckIII in flies) at distinct interaction sites (diagrammed in Figure 5B). Consistent with STRIPAK’s role as a negative regulator, these binding interactions are phosphorylation dependent, indicating that only activated STE20 kinases are recruited to the STRIPAK complex.

The roles of other STRIPAK components are less understood. An intact STRIPAK complex is required for negative regulation of Hippo signaling in distinct *Drosophila* cellular contexts, but the contribution of Mob4/Phocein has yet to be defined (Ribeiro et al., 2010; Schulte et al., 2010; Sakuma et al., 2016; Zheng et al., 2017; Neisch et al., 2017; Gil-Ranedo et al., 2019). Mob4/Phocein proteins have been shown to bind directly to STE20 kinases in both fly and mammalian cells (Ribeiro et al., 2010; Couzens et al., 2017; Chen et al., 2018), but it is unclear if Mob4/Phocein is required to recruit GCK-type STE20 kinases to the STRIPAK complex. To function as a recruiting protein, Mob4/Phocein must function as an adaptor between its STE20 kinase binding partner and one or more STRIPAK components. However, the nature of the Mob-STRIPAK binding interaction is unknown, even though the founding member of the Class IV/Phocein Mobs was identified as a rat Striatin-binder using yeast two-hybrid screens (Baillat et al., 2001; see also Gordon et al., 2011). Alternatively, Mob4/Phocein proteins may contribute to STE20 kinase silencing through competitive interactions with Class I Mobs (Figure 5C). By blocking Class I Mob access to activating STE20 kinases, Mob4/Phocein proteins would prevent formation of a Warts/LATS ternary activation complex (Figure 5A). Many open questions remain about Mob participation in STRIPAK complexes and their contributions to STRIPAK-dependent negative-regulation of Hippo-like pathways.

## Concluding Remarks

Most animal Mob studies have focused on their roles as NDR kinase activators, now well-established by combinations of genetic, cell biological, and biochemical approaches. Potential roles independent of Hippo and Hippo-like pathways exist, and remain to be explored further (Hergovich et al., 2006b; Hergovich, 2016). Our expanded understanding of animal Mobs has largely come from the research enterprise investigating the Hippo growth regulation pathway. However, as new cellular contexts for Hippo signaling or NDR kinase activation are uncovered, the question of whether Mobs are involved is increasingly overlooked. The role of Mobs in full activation of Warts/LATS and Tricornered-like kinases would provide a tool to explore whether alternative STE20 kinases result in weaker activation of the downstream NDR kinases.

Even the roles of Mobs as NDR kinase activators need to be clarified. Class I Mobs are demonstrated co-activators of Warts/LATS NDR kinases in flies, mice, and humans, with defined binding interactions. However, Class II Mob functions in NDR kinase regulation remain unclear. Fly and mammalian Class II Mobs have been shown to bind exclusively to Tricornered-like NDR kinases, but currently there is no consensus concerning the nature of these interactions. Some studies suggest that Class II Mobs promote activation of Tricornered-like kinases, others suggest an inhibitory function. Furthermore, the roles of Class I and Class II Mobs are increasingly overlooked in studies of both Hippo kinase signaling and inputs into the Hippo signaling pathway from alternative GckIII and MAP4K kinases.

Localization of Hippo signaling components to distinct membrane domains is a recurring theme in polarized cell types (Fulford et al., 2018). In at least one case, localization is coincident with competitive binding interactions that prevent binding of the Mob1 activator for Warts/LATS kinase (Dutta et al., 2018). However, other studies that investigate differential localization of activated Warts/LATS kinase do not test for involvement of a Mob partner. Another study suggests that Mob activation is not essential as the first step for Warts/LATS activation, but that it is necessary to fully activate the kinase (Mana-Capelli and McCollum, 2018). Whether activation by a Mob can be bypassed remains an open question that becomes more important, as more alternative pathways to activate Warts/LATS and Tricornered-like kinases are uncovered.

Early studies of Class 1 Mobs in yeast and flies demonstrated an essential role in activation of their partner NDR kinases. While current models suggest a more flexible requirement, this activity raises the question of whether the availability of Mob adaptors is regulated within cells, either through transcriptional or post-transcriptional mechanisms, and whether Mob protein activity is regulated independently from their partners. These questions about regulation of Mob levels have been best studied in mouse and human systems (Sasaki et al., 2007; Lignitto et al., 2013; Otsubo et al., 2017; Chen et al., 2018; Kim and Jho, 2018a), as covered recently in Gundogdu and Hergovich (2019).

Phylogenetic comparisons indicate that Mobs exhibit functional diversity, paralleled by sequence diversity of animal Mob classes. Multiple experimental approaches suggest that different animal Mob classes have partially overlapping, yet distinct binding partners, many of which are conserved across eukaryotes. This review focused on those conserved core functions. Class I Mobs are phosphorylated by Hippo-like kinases, but are they phosphorylated by other STE20 kinases? Are Class II Mobs also phosphorylated by Hippo kinases or others? Class II Mobs bind exclusively to Tricornered-like NDR kinases, but we lack a consensus view of the functional consequences for these interactions. Does the lack of consensus reflect a context-dependent difference, or differences in the types of assays used?

Proteomic studies of mammalian Class III and IV/Phocein Mobs suggest that these proteins do not bind NDR kinases. In contrast, Class IV/Phocein Mobs are essential components of the STRIPAK complex that antagonizes Hippo signaling in animals, a role also implicated by genetic studies of filamentous fungi (Shomin-Levi and Yarden, 2017; Kuck et al., 2019). In spite of this conserved requirement across model organisms, we know little about the molecular aspects of Mob4/Phocein function within STRIPAK. Mob4/Phocein proteins stand out as a potential regulatory node, through these Mobs’ ability to associate with both STRIPAK-associated phosphatase and GCK-type STE20 kinases. What is the nature of the STE20 kinase association with STRIPAK, is it purely a phosphatase substrate or does it have a different role? The role of Class IV/Phocein Mobs in recruiting substrates to STRIPAK for dephosphorylation is an open question.

Identification of Class IV/Phocein Mobs and STRIPAK complex components in filamentous fungi raises questions about the apparently divergent regulation of Hippo-related STE20 kinases in unicellular yeasts, *S. cerevisiae* and *Schizosaccharomyces pombe*. These yeasts have a homolog of phosphatase PP2A, but not of other protein components of the STRIPAK complex, such as Striatin. Kuck et al. (2019) point to alternative yeast proteins that may substitute as PP2A-STE20 kinase linkers instead. These distinctions raise questions about divergence between unicellular and multicellular eukaryotes, and whether switching between upstream kinases to activate NDR kinases may be particularly important in organizing multicellular structures, such as the fruiting bodies of filamentous fungi (Beier et al., 2016). In this regard, it is striking that the ciliate *Tetrahymena* appears to have a Mob4 homolog (Soares et al., 2019). To understand the broadly conserved network of Mob functions in Hippo and Hippo-like pathways, it will be valuable to investigate whether ciliates, such as *Paramecium* and *Tetrahymena*, have STRIPAK complexes that regulate switching between the ciliate NDR kinase pathways.

We have emphasized the core functions of Mobs that are conserved across eukaryotes in this review; others have focused on the diverse roles of the numerous mammalian Mobs in distinct pathologies (Gundogdu and Hergovich, 2019). Additional functions for NDR kinases and Mobs are just beginning to show up from unbiased screens in distinct cell types, whether based on traditional genetics, RNAi, chemical genetics or protein-protein interactions. The potential for cross-regulation and/or competition between Warts/LATS kinases and Tricornered-like kinases is apparent through their regulation by shared Class I Mob and Hippo activators but is only beginning to be investigated in detail (Zhang et al., 2018). Our understanding of both Mobs and Hippo pathway signaling would benefit from experiments to directly test whether a Mob-NDR kinase partnership and STRIPAK antagonism influence the pathway output in each new cellular context.

## Author Contributions

This manuscript is adapted from the dissertation authored by JD, in partial completion of his Ph.D. degree. LR contributed to writing of this manuscript.

## Conflict of Interest

The authors declare that the research was conducted in the absence of any commercial or financial relationships that could be construed as a potential conflict of interest.

## Abbreviations

AGC: protein kinase AG and C-related
Dbf2: Dumbbell former protein 2
GCK: germinal center kinase
LATS: large tumor suppressor
MAP4K: mitogen activated protein kinase kinase kinase kinase
Mats: Mob as tumor suppressor
MEN: mitotic exit network
Mob: Mps1 binder-related
MOR: morphogenesis Orb6 network
MST: Mammalian STE20-like
NDR: nuclear Dbf2-related
NTR: N-terminal regulatory
PAK: p21-activated kinase
PP2A: protein phosphatase 2A
RAM: regulation of Ace2p and morphogenesis
RNAi: RNA interference
SARAH, Salvador: Rassf and Hippo
SIN: septation initiation network
SLMAP: sarcolemma associated protein
STE20: Sterile-20
STK: serine/threonine kinase
STRIP: striain interacting protein
STRIPAK: striatin interacting phosphatase and kinase
WASP: Wiskott–Aldrich syndrome protein.
